# Anatomy and Physiology

**Published:** 1838-04

**Authors:** 


### PART THIRD.

### Selections from the Foreign Journals.

---

### ANATOMY AND PHYSIOLOGY.

### *On the Structure of the Iris in Birds, and the mechanism by which it is moved.* By Dr. August Krohn, of Petersburg.

Dr. Krohn considers the iris of birds as composed of four principal coats; 1st the anterior layer of pigment; 2d, the fibrous layer; 3d, a delicate membrane serving as support to the fibrous coat; and 4th, the posterior layer of pigment or the uvea.

1. On the anterior pigment depends the great variety of shades of colour of the iris. Of the light tints the white and yellow occur most frequently, but the dark also occur in every shade from light brown to the deepest black. In a few cases, as in the white goose, the anterior pigment is wanting, and the iris then assumes a blue colour caused by the dark uvea being seen through the colourless iris. The yellow colour of the iris in owls and in the common fowl is owing to the presence of a yellowish oil which is contained in small vesicles, and is not, at least in some owls, at all dependent on the molecules of the pigment. In other cases it appears as if the yellow colour were owing to a combination of the oil with a yellowish pigment. Dr. Krohn is doubtful whether an extremely fine membrane coats the anterior pigment, to preserve it from the action of the aqueous humour.

2. The following results, regarding the nature of the fibrous coat, were obtained from an examination of the iris in—*(Birds of Prey:)* Strix Bubo, Str. nisoria, Falco ossifragus; (*Climbing Birds:*) Psittacus erithacus; (*Singing Birds:*) Panus ater, Fringilla domestica, Fr. spinus, Fr. enucleator, Loxia pyrrhula; *(Gallinaceous Birds:)* Phasianus Gallus, Tetrao Bonasia, Meleagrus Gallopavo, Coturnia dactylisonus, Columba œnas; (*Swimming Birds:*) Anas anser, Anas Boschas (domestic.)

The whole surface of the iris, from the ciliary border to the pupil, is covered with a large number of fibres running concentrically with the pupil, but requiring the microscope to be accurately distinguished. They are particularly distinct towards the ciliary border, and then appear to be composed of several layers heaped on each other; some of them deviate in a slight degree from the true circular course, and by crossing with others produce in some places an interweaving of fibres. Towards the pupil the course of the fibres is more parallel, and their thickness diminishes progressively as they approach that aperture. Below the microscope these fibres present all the characters of muscular fibres; they appear as transversely marked cylinders, are easily torn, both in a longitudinal and transverse direction, and their transverse lines become more distinct by maceration in alcohol, and thus the fibre in some measure acquires a resemblance to the trachea of insects. It is impossible to determine, owing to the small field of vision of the microscope, whether the single fibres form entire circles, but at any rate it is certain that they belong to the smallest fibres of the muscular tissue; in diameter the largest of them correspond most nearly with the fibres of the heart, but their breadth seems to vary with the class and species of the bird. It is remarkable that in owls the iris is not, as in other birds, in immediate contact with the ciliary processes, but is connected to them solely by the vessels which stretch through the aqueous humour from the choroid to the iris. In order to examine the muscular fibres both layers of pigment must be removed by means of a hair pencil and frequent agitation of the iris in water; a small piece should then be excised, and subdivided beneath the field of the microscope by small needles.

3dly and 4thly. The thin delicate membranes posterior to the muscular layer, and the posterior layer of pigment present nothing remarkable. The latter, like the anterior layer is probably covered by a thin delicate coat, to preserve it from being washed off by the aqueous humour.

Dr. Krohn has likewise devoted two or three pages to a description of the blood-vessels and nerves of the iris, with reference particularly to some peculiarities in their distribution in the owls; but as his remarks are scarcely susceptible of abridgment we are forced to pass them over.

The reader will scarcely doubt, says Dr. Krohn, that the movements of the iris and its frequent oscillation depend upon the expansion and contraction of the circular fibres of the muscular layer; they contract to diminish the aperture of the iris, and the dilatation of the pupil is owing simply to their relaxation. In this respect the iris corresponds with the other sphincter muscles, but there is this difference, that the contraction or dilatation of the pupil varies rapidly owing to the frequent change of the force, and the peculiar nature of the stimulus. A circular muscle, a sphincter pupillæ, is therefore sufficient to account for all the changes in diameter in the pupil of living animals; and with this view the experiments of Mayo seem to correspond. According to this physiologist, the division of the N. oculomotorius within the skull of living pigeons was followed by immovability and dilatation of the pupil, or in other words by paralysis of the sphincter pupillæ; whilst mere irritation of the nerve produced a contracted pupil, in the same way as mechanical irritation of a nerve supplying any muscle is followed by contraction of that muscle.

Dr. Krohn next proceeds to speak of the circle of muscular fibres, first described by Crampton, which lie on the inner surface of the osseous plates. These fibres arise from the osseous circle, and are inserted, according to Crampton, into the inner surface of the cornea; according to Mannoir into the ciliary border of the iris; and according to Dr. Krohn, into the anterior part of the upper wall of the canal of Fontana. Examined with the microscope they present the same appearances as the fibres of the iris; and are identical in structure. According to Crampton they serve to flatten the cornea and to adjust the eye for seeing distinctly at great distances; but Dr. Krohn does not acquiesce in this use of the muscle, although he confesses he cannot offer any probable conjecture as to its action. According to Mannoir's view it would act as a dilator of the pupil; but it is very improbable that the fibres are inserted into the iris.

The preceding observations refer only to the eyes of birds; the examination of the eyes of the mammalia requires greater care and is more difficult: Dr. Krohn has, however, found in the human iris a layer of fibres running concentric with the pupil, but he is not yet prepared to speak decidedly as to their nature.

*Müller's Archiv. Jahrgang,* 1837. Heft iii.-iv.

---

### *On the Nature of the Degeneration of the Kidney in Bright's Disease.* By Dr. Gluge.

In this disease the cortical substance of the kidney is paler, and the tubular substance redder than in the healthy state, and the former acquires a greater development at the expense of the latter. The cortical substance has lost its smoothness and has become granular, and this granular appearance is also seen on the surface of the kidney after removal of the tunica propria. Is this granular appearance owing to the deposition of a foreign substance? In order to ascertain this point a drop of fluid from the cortical substance was examined with the microscope and found to contain a large quantity of albumen, a large number of peculiar globules, detected by Dr. Gluge in inflammatory secretions and termed by him inflammatory globules, and a few globules of pus. Fluid pressed from the tubular substance likewise contained inflammatory globules, but in infinitely smaller quantity. On examining a thin segment of the cortical substance with the microscope these globules were found deposited in immense numbers between the urinary ducts; they were likewise found in the medullary substance but in much smaller quantity. Dr. Gluge therefore concludes that the degeneration of the kidney in Bright's disease consists in inflammation of the cortical substance of the kidney, which extends, though in a comparatively slight degree, to the tubular substance.

*Wochenschrift für die gesammte Heilkunde. Jahrgang*, 1837. No. 39.

---

### *On the Existence of the Polystoma Sanguicola in the Human Blood.* By Dr. DELLE CHIAJE.

CASE I. Was of a man, æt. twenty-five, subject for some time to næmoptysis. His temperament was sanguine, his parents were robust, but he had been suckled by a nurse affected with syphilis. He led a dissipated life, had had syphilis and itch. The hæmoptysis occurred first in an alarming manner, after he had taken a bath in the sea, during the heat of summer. Expectoration continued notwithstanding the means which were employed. On one occasion the patient was seized with an attack of hæmoptysis in the presence of his physician, Dr. Gallo. On examining the blood half an hour afterwards, Dr. Gallo found some small flattened worms, resembling little leeches, which were attached to the sides of the basin. The parents assured him that they had observed similar animals every time that an attack of hæmoptysis had occurred. The patient became phthisical, and Dr. Gallo had no further opportunity of observing the matters expectorated.

CASE II. M. Tolinea observed a similar case of hæmoptysis, in an individual who had been previously affected with syphilis and scabies. Three of the Polystoma sanguicola were found in the blood expectorated. From this period, the patient was given into the charge of Dr. Delle Chiaje, but here also the disease rapidly passed into phthisis, and the author was disappointed in his endeavours to preserve alive these remarkable entozoa.

The author regards the two preceding cases together with others of a more ancient date, (e. g. that of Borelli in his Obs. Cent. iii. Obs. iv.,) as irrefragable proofs of the existence of the polystoma, although it has been denied by the majority of naturalists. Dr. Delle Chiaje endeavours to dissipate all doubt on the subject by supposing that the polystoma does not exist in the blood itself; according to his view, it is lodged in the pulmonary parenchyma in the same manner as the diastoma hepaticum occupies the parenchyma of the liver, the tetrastoma, the papillæ of the kidneys, and the polystoma pinguicola, the ovaries. It is not until after they have perforated the walls of vessels that these animals enter the circulation.

The author thus describes the polystoma. It resembles at first sight, a drop of coagulated blood, flattened like the pips of a gourd, but of a deeper red colour than that of the blood which surrounds it. When touched, this seeming drop of blood moves distinctly. The animal has two extremities, the one pointed, the other rounded; in its contracted state its length is three lines, and its breadth two lines. When it stretches itself, its length is ten lines and its breadth three lines. Its structure, which is almost annular, may then be distinguished. It executes its movements by a double extension, in the same manner as leeches. The animal reacts against the least impression, and passes immediately from rest to a motion more and more quickened, according as it is found swimming in a fluid or attached to the sides of a vessel. *Osservatore Medico di Napoli.*

---

### *On the existence of the Ovum at a very early period in the Female Economy, and on the consequent necessity of admitting a new period in the History of Human Development.* By Dr. C. G. CARUS, of Dresden.

BY the labours of preceding physiologists the axiom had been established, "that man and the mammalia derive their origin from an ovum existing in the ovary previous to impregnation," but there were wanting data to establish at what period of life these ova first become developed in the ovaries of man and of the mammalia. The investigation of this point forms the subject of the paper before us.

Dr. Carus with a little practice was soon able to pick out the Graafian vesicles from the ovaries of very young calves, and to submit them to examination beneath the field of the microscope. The extracted vesicle was carefully laid open with a cataract needle, and a little attention then sufficed to distinguish the ovum, surrounded by the discus proligerus, swimming about in the fluid which escaped. The chorion, the yolk, the germinal vesicle with the cicatricula were all remarkably distinct.

The opportunities of examining the ovaries of female infants were more difficult to be obtained; but in the spring of 1837, the opportunity of examining the ovary of an infant four days old presented itself. In this case it was impossible to detect a Graafian vesicle already filled with fluid and containing an ovum, but on submitting thin segments of the ovary, subjected to moderate pressure according to the method of Purkinje and Valentin, to examination, there was no difficulty in perceiving ova of various sizes, characterized by the yolk and germinal vesicle, but closely enclosed by the Graafian vesicle and substance of the ovary.

The examination of the ovary of an infant, one and a half years of age, showed considerable differences. In this case a number of the Graafian vesicles had expanded to the breadth of a quarter or even half a line. The child had been affected with rhachitis, and the circulation had in consequence been obstructed, and blood even been effused into some of the vesicles. In others the ova were found in a semi-dissolved state; but in one of the largest the ovum was found quite entire and distinct, and in another there was found an imperfectly developed ovum, in which the clear circle of albumen, between the membrane of the yolk and the chorion, and part of the yolk were discernible. Dr. Carus here calls attention to the pathological conditions of the ova, as above alluded to, as throwing light upon the origin and cause of dropsy and degeneration of the ovaries, and as affording an explanation of one of the causes of sterility.

In examining the ovaries of a girl, æt. four years and a half, who had died of pneumonia, both ovaries were found to contain one perfectly developed Graafian vesicle six-eighths of a line in diameter, each of which contained an ovum of the one-twelfth of a Vienna line in diameter, with yolk, germinal vesicle, and cicatricula all perfectly distinct, swimming in a clear albuminous fluid. Besides these large vesicles both ovaries contained a number of smaller ova from one-sixtieth to one-fourteenth of a Vienna line in diameter.

From these observations Dr. Carus concludes: 1stly, that the ova, the germs of future individuals, are already formed before the birth of the female, so that in the last period of pregnancy three generations exist in the body of one individual; 2dly, that shortly after the birth of the female, at least before the end of the first year, several of the Graafian vesicles become developed to such a degree around the ova, as to present an appearance not materially different from their condition at the period of puberty, and consequently, it is rendered probable that the only obstacle to impregnation at an early age lies in the structure of the external parts. 3dly, that the matured human ovum, when it has in some measure been isolated from the maternal parts by the development of the Graafian vesicle and its fluid contents, may remain in a state of latent life for an indefinite number of years till roused into activity by the stimulus of impregnation. 4thly, that a new period, that of the latent life of the ovum, must henceforward be adopted in the history of the development of man.

Dr. Carus concludes his paper with some comparative remarks on the latent life of the human ovum, and the ova of plants and insects. It is well known that the seeds of plants may retain their latent life for thousands of years, but this quality is essentially different from the latent life of the human ovum, as in the latter there has been no fructification, and because the ova of plants before fructification are incapable of latent life. The eggs of the articulated animals are also incapable of remaining for any length of time in a state of latent life, although after fructification has taken place, but before there is any appearance of the embryo, they may remain, often for a long period, in a latent state. The same kind of latent life is apparent also in the impregnated eggs of the higher amphibia and of birds, when the egg has reached a certain stage of its development. The long latent life of the human ovum Dr. Carus considers as one of the consequences of the high destiny of man, as affording time for important but imperceptible changes in the structure of the germ. *Müller's Archiv. Jahrgang*, 1837. Heft iv.

### PATHOLOGY, PRACTICAL MEDICINE, AND THERAPEUTICS.

